# Correction: Svertoka et al. Decision Support Algorithm Based on the Concentrations of Air Pollutants Visualization. *Sensors* 2020, *20*, 5931

**DOI:** 10.3390/s25082556

**Published:** 2025-04-18

**Authors:** Ekaterina Svertoka, Mihaela Bălănescu, George Suciu, Adrian Pasat, Alexandru Drosu

**Affiliations:** 1 Department of Telecommunications, University Politehnica of Bucharest, 061071 Bucharest, Romania; esvertoka@elcom.pub.ro; 2 Department of Telecommunications, Brno University of Technology, 61600 Brno, Czech Republic; 3 Beia Consult International, 010158 Bucharest, Romania; mihaela.balanescu@beia.ro (M.B.); adrian.pasat@beia.ro (A.P.); alex.drosu@beia.ro (A.D.)

Certain citations included in the manuscript were not fully aligned with the scope of the study. These citations were incorporated following recommendations made by a reviewer during the peer-review process. The Academic Editor and the authors have agreed to update the following aspects of this publication [1]:

## Review Report Correction

Reviewer Report 2 was removed from the peer-review record (https://www.mdpi.com/1424-8220/20/20/5931/review_report (accessed on 20 March 2025)).

## Text Correction

In Section 2, Paragraph 1, the sentence “However, a number of challenges are still present in this area, such as a need for more accurate monitoring, finding the trade-off between the functionality of a device and its form factor, power consumption, and price [10].” was removed.

In Section 2, Paragraph 2, “Representative work can be observed in [11] where a complete fusion framework for medical-related data is presented. As the diversity of medical sensors increases, it is mandatory that certain ways of managing different data to be developed, exactly as in [11], where the effectiveness of the proposed method is also explored.” was removed.

## References Correction

References [10,11] were removed. With this correction, the order of some references has been adjusted accordingly.

The authors state that the scientific conclusions are unaffected. This correction was approved by the Academic Editor. The original publication has also been updated.

## References

[1] Svertoka E., Bălănescu M., Suciu G., Pasat A., Drosu A. (2020). Decision Support Algorithm Based on the Concentrations of Air Pollutants Visualization. Sensors.

